# Effect of Twin Boundary Motion and Dislocation-Twin Interaction on Mechanical Behavior in Fcc Metals

**DOI:** 10.3390/ma13102238

**Published:** 2020-05-13

**Authors:** Jaber Rezaei Mianroodi, Bob Svendsen

**Affiliations:** 1 Microstructure Physics and Alloy Design, Max-Planck-Institut für Eisenforschung, 40237 Düsseldorf, Germany; bob.svendsen@rwth-aachen.de; 2 Material Mechanics, RWTH Aachen University, 52062 Aachen, Germany

**Keywords:** twin boundary, molecular dynamics, dislocations, mechanical response

## Abstract

The interplay of interface and bulk dislocation nucleation and glide in determining the motion of twin boundaries, slip-twin interaction, and the mechanical (i.e., stress-strain) behavior of fcc metals is investigated in the current work with the help of molecular dynamics simulations. To this end, simulation cells containing twin boundaries are subject to loading in different directions relative to the twin boundary orientation. In particular, shear loading of the twin boundary results in significantly different behavior than in the other loading cases, and in particular to jerky stress flow. For example, twin boundary shear loading along 〈112〉 results in translational normal twin boundary motion, twinning or detwinning, and net hardening. On the other hand, such loading along 〈110〉 results in oscillatory normal twin boundary motion and no hardening. As shown here, this difference results from the different effect each type of loading has on lattice stacking order perpendicular to the twin boundary, and so on interface partial dislocation nucleation. In both cases, however, the observed stress fluctuation and “jerky flow” is due to fast partial dislocation nucleation and glide on the twin boundary. This is supported by the determination of the velocity and energy barriers to glide for twin boundary partials. In particular, twin boundary partial edge dislocations are significantly faster than corresponding screws as well as their bulk counterparts. In the last part of the work, the effect of variable twin boundary orientation in relation to the loading direction is investigated. In particular, a change away from pure normal loading to the twin plane toward mixed shear-normal loading results in a transition of dominant deformation mechanism from bulk dislocation nucleation/slip, to twin boundary motion.

## 1. Introduction

Among deformation mechanisms in (higher symmetry) metals, perhaps the most prevalent are dislocation-based or -related ones. Besides dislocation glide, these include for example dislocation-mediated twinning, and dislocation-mediated phase transformations. A prominent example of the former is twinning induced plasticity (TWIP), and of the latter, transformation induced plasticity (TRIP). TWIP steels are particularity interesting for industrial applications due to their high ductility and strength [1,2]. Dislocations are modeled at different length and time scales, ranging from continuum scale, mean field descriptions through dislocation density [3] or phase-field [4,5] to mixed atomistic-continuum methods such as atomistic phase-field [6,7] as well as full atomistic models such as molecular dynamics (MD). Interaction between dislocations and other defects, such as interfaces and twin boundaries, influences the ductility and hardening of material. Investigating individual dislocations and their interaction with individual microstructural constituents, such as twin boundaries, provides further insight into processes such as hardening at larger scales.

In a number of previous works, MD simulations have been employed to investigate interaction between dislocations and twin boundaries. For example, Sedlmayr et al. [8] studied twinning and the resulting stress-strain behavior in Au nanowhiskers via both scanning electron microscopy and MD simulation. They identified two types of twin formation in their samples, one with a large number of small twins, and the other with one long twin, resulting in different stress-strain behavior. Indeed, whereas the former results in generally smooth, slowly varying stress-strain behavior, this behavior in the latter case is fluctuating or “jerky”. Cheng and Ngan [9] investigated the deformation behavior of colliding Cu nanoparticles via MD simulation. They showed that twinning is the dominant deformation mechanism in “smaller” particles (especially at higher loading rates), whereas larger particles generally deform through dislocation glide. Focusing on single or few twin boundaries, Jin et al. [10,11] studied the interaction of bulk screw and mixed dislocations with twin boundaries in a number of fcc metals such as Al, Cu and Ni. Depending in particular on loading conditions, they found that dislocations either transmit across, or glide along, the twin boundary. The results of Hu et al. [12] imply that twin boundary motion under shear loading (resulting in twinning or detwinning) depends on the angle of the shear direction relative to 〈110〉 on {111} planes. Li et al. [13] employed in situ high resolution transmission electron microscopy to study interaction of gliding dislocations with coherent twin boundary in Cu. They observed multiplication of partial dislocations as they react with the twin boundary, resulting in translation of the boundary. Seo et al. [14] examined the deformation behavior of defect free Au nanowires due to twin boundary motion and propagation. Twin boundary propagation resulted in crystal reorientation from [110] to [100]. The stress-strain curves show initial elastic behavior followed by perfect plastic behavior until the twin boundary is fully propagated. After crystal reorientation, the sample behaves elastically.

Liebig et al. [15] performed micro-compression tests on twinned pure Cu and α-brass specimens with lower stacking fault energy. They carried out the experiments in multiple orientations including [112], [110] and [259]. The stress-strain results of twinned specimens under vertical loads in [112] and [110] directions show minor deviation from the single crystal samples. Total lack of dislocation pile-up due to low slip transfer barrier, is claimed to contribute to a small twin boundary effect in these loading cases. In contrast, compression loading in [259] direction results in strong pile-up at the twin boundary having a strong effect on the stress-strain behavior. In comparison to pure Cu, brass samples with a lower stacking fault energy (and so lower probability of cross slip) exhibit a more serrated flow stress since the plastic strain is accommodated by fewer dislocation systems. Zhao et al. [16] employed molecular dynamics to study deformation mechanism in nanotwinned Cu with different twin orientations. In agreement with the current results, their results exhibit a transition in dominant deformation mechanism from (i) bulk dislocation slip to (ii) twin boundary motion (twinning, detwinning) to (iii) twin boundary motion and bulk slip/slip transfer to (iv) bulk slip as the angle between the twin plane normal and loading direction increases from 0 to 90 degrees. More recently, Zhang et al. [17] studied the mechanical response of coherent twin boundaries under uniaxial tension and compression as well as under in-plane shear loading. They compared perfect and defective twin boundaries in Cu at temperatures of 100 K, 300 K and 600 K. Little efffect of the twin boundary on dislocation sources was observed by them under uniaxial normal loading. Under shear loading, they observed stick-slip behaviour characterized by a saw-tooth-like variation of stress with time or “jerky” flow.

One purpose of the current work is to gain further insight into the underlying atomic and deformation mechanisms behind such jerky flow related to twin boundary motion. A second purpose is the investigation of the interaction between twin boundary motion and bulk dislocation slip on the resulting mechanical behavior of twinned fcc metals. As in most of the studies discussed above, MD simulations are employed for this purpose here. After discussion of the corresponding simulation set-ups and cases studied in Section 2, the work turns in Section 3 to a presentation and discussion of simulation results. In particular, this includes the stress-strain behavior of untwinned and twinned crystals subject to different loading conditions and elucidation of the underlying atomic and deformation mechanisms in Section 3.1. To gain further insight into the case of twin boundary motion via nucleation and glide of twin boundary partial dislocations, the velocity of these and the corresponding energy barriers to glide are determined in Section 3.2 and compared with their bulk counterparts. This is followed in Section 3.3 by a discussion of results for the dependence of deformation mechanism on twin boundary orientation. In both cases, the dependence of atomic and deformation mechanisms on external loading conditions, and the concomitant stress-strain behavior, are documented in detail. The work ends with a summary and discussion in Section 4.

## 2. Simulation Set-Up and Cases Studied

In this work, the software LAMMPS [18] is employed for all MD simulations, and the software Ovito [19] for all visualizations and post processing. MD simulation results for Cu (lattice constant $a_{0}=3.615$ Å) are based on the embedded atom (EA) potential from [20]. As shown by Ezaz et al. [21] and Boyer et al. [22], this potential reproduces stacking fault energy values close to those obtained from quantum density functional theory, making it a good choice for twinning and dislocation simulations.

### 2.1. Simulation Cell Initialization and Loading Conditions

All simulation cells employed here are fully periodic and triclinic. To initialize these for all simulation cases, the cell energy is first minimized via damped dynamics [23] in 5000 time steps of 2 fs. This is followed by relaxation of all stress components to zero in the NPT (i.e., Parrinello-Rahman [24]) ensemble at 300 K in 10,000 steps. Lastly, the temperature is reduced in another 10,000 steps to 20 K at zero stress. From this initial state, simulation cells are loaded at a constant strain rate of $10^{9}$ s^−1^ (via the “box deform” command in LAMMPS) for 100,000 time steps of size (Simulations were also carried out for Δt=4 fs to verify independence of the results from this choice.) Δt=2 fs in the NPT ensemble at 20 K. All stress components are set to zero in the NPT ensemble during loading except the stress component corresponding to the applied strain. Thermostat and barostat damping coefficients are set to 50 and 100 times the time step size, respectively, in all simulations.

### 2.2. Loading Cases

**Figure 1:**
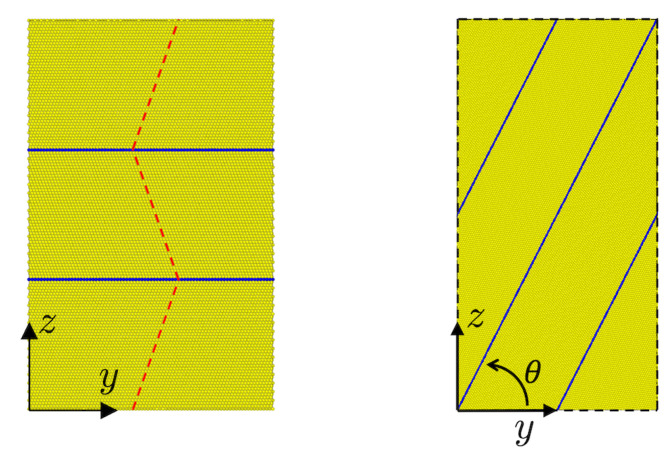
Simulation cells used to study deformation mechanisms and stress behavior in the presence of twinning. (**Left**) cell employed for comparison with the untwinned case. (**Right**) cell employed for variable twin boundary orientation. Visualization based on common neighbor analysis. Yellow: lattice (fcc). Blue: twin boundary (hcp). See text for discussion.

The effect of twinning on stress-strain behavior is studied in the current work with the help of both untwinned (i.e., perfect), and twinned, fcc simulation cells. Figure 1 displays two different twin configurations employed in this work.

In particular, comparison with the untwinned case is carried out for the twin configuration in Figure 1 (left) in what follows. This simulation cell has dimensions of $\left(L_{x},L_{y},L_{z}\right)=\left(35\sqrt{2}a_{0},20\sqrt{6}a_{0},40\sqrt{3}a_{0}\right)$ and contains 756,000 atoms. The *x* and *y* directions are parallel to $\left[\bar{1}10\right]/\sqrt{2}$ and $\left[\bar{1}\bar{1}2\right]/\sqrt{6}$ ($\left[1\bar{1}0\right]/\sqrt{2}$ and $\left[11\bar{2}\right]/\sqrt{6}$), respectively, in the untwinned (twinned) regions, and *z* is parallel to the twin boundary normal $\left[111\right]/\sqrt{3}$.

The effect of variable twin boundary orientation relative to the loading direction (*z*) is studied with the help of the simulation cell shown in Figure 1 (right).

All twin boundaries considered in this work are coherent. The angle θ represents the angle between the twin plane and the xy plane of the simulation cell as shown. As well, θ is the angle between twin plane normal and the loading direction *z* in what follows. In particular, θ values of 0, 15, 30, 45, 60, 75 and 90° are considered here. With respect to θ and loading in the *z* direction (i.e., $T_{zz}$), $\theta =0^{{}^{\circ}}$ for example corresponds to pure normal loading, $\theta =45^{{}^{\circ}}$ to mixed normal-shear loading, and $\theta =90^{{}^{\circ}}$ to pure parallel, loading. The initial twin spacing is set to $15\sqrt{3}a_{0}$ in all the cases. The simulation cell size is adjusted in each case to satisfy full periodicity as well as defect free closure of the twinned regions. For example the cell shown in Figure 1 (right) has a size of $\left(L_{x},L_{y},L_{z}\right)=\left(173.4,307.4,15.3\right)$ Å for twin angle $\theta =60^{{}^{\circ}}$.

### 2.3. Dislocation Velocity

**Figure 2:**
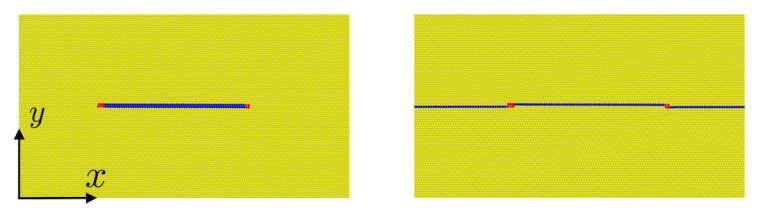
Dislocation configuration in a periodic simulation cell for determination of the velocity of bulk (**left**) and twin boundary (**right**) straight partial dislocations. Left: partial edge dislocation dipole in the bulk. Right: partial edge dislocation dipole on the twin boundary. In all cases, the dislocation line is parallel to the *z* direction $i_{z}$, the glide plane unit normal to the *y* direction $i_{y}$, and the glide direction to the *x* direction $i_{x}$. The cell size is $\left(L_{x},L_{y},L_{z}\right)=\left(60\sqrt{6}a_{0},48\sqrt{3}a_{0},3\sqrt{2}a_{0}\right)$ in the edge, and $\left(L_{x},L_{y},L_{z}\right)=\left(120\sqrt{2}a_{0},48\sqrt{3}a_{0},3\sqrt{6}/2a_{0}\right)$ in the screw case.

To gain additional insight into twin boundary motion resulting from twin boundary dislocation nucleation and glide, the velocity of both interface and bulk partial edge and screw dislocations are determined here. The corresponding periodic cell and dipole configurations are shown in Figure 2.

As well-known (e.g., [25]), dislocation motion is driven by the Peach-Köhler force f=Tb×l, with l the dislocation line direction, b the Burgers vector, and T the stress tensor. The glide component $f_{\mathrm{gl}}=m\cdot f=b\cdot Tn$ of f is that parallel to the (in-plane) dislocation line unit normal m:=n×l, with n the glide plane unit normal. In particular, for the simulation set-ups in Figure 2, $\left(m,n,l\right)=\left(i_{x},i_{y},i_{z}\right)$, $f_{\mathrm{gl}}=bT_{xy}$ for the edge case b=bm, and $f_{\mathrm{gl}}=bT_{yz}$ for the screw case b=bl. Consequently, all stress components are zero in the simulation using NPT ensemble except $T_{xy}$ for the edge, and $T_{yz}$ for the screw, case, which are set to 1 GPa. Reported velocities are monopole velocities. The system size is chosen to minimize dipole and periodic interaction effects. This was confirmed by doubling the cell sizes in *x* and *y* while keeping the size in *z* (dislocation line direction) the same, resulting in a velocity difference below 5%.

## 3. Results and Discussion

All stress results to follow represent the virial stress of the simulation cell. Since the temperature is relatively low, the kinetic contribution to stress is negligible. Strain results to follow represent the linear cell strain $E_{ij}=1/2\left(\partial_{i}u_{j}+\partial_{j}u_{i}\right)$, with $u_{i}$ the cell displacement vector. Zero cell displacement corresponds to zero cell stress.

### 3.1. Untwinned and Twinned Stress-Strain Behavior for Different Loading Cases

**Figure 3:**
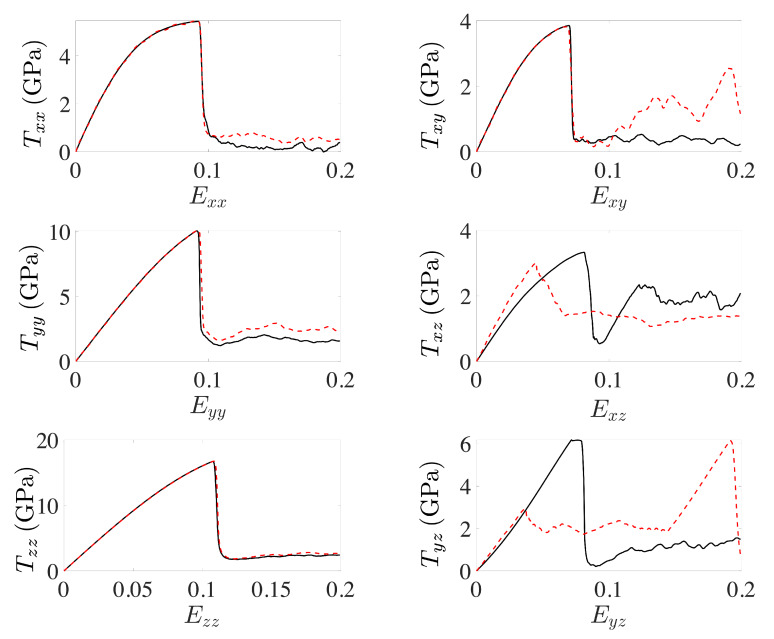
Stress-strain curves for untwinned (solid black line) and twinned fcc crystal (dashed red line) subject to loading with respect to a single stress component. All other components are zero.

In this section, stress-strain results are obtained and discussed for the simulation set-up and loading cases described in Section 2.2. Figure 3 shows stress-strain results for the loading of untwinned and twinnned single crystal Cu based on the simulation cell in Figure 1 (left).

As shown in the left column, the stress-strain behavior is similar in the three normal loading cases $T_{xx}$, $T_{yy}$, $T_{zz}$. In each case, elastic lattice deformation and stress increase is followed by bulk dislocation nucleation, stress relaxation and dislocation glide during continued loading. The twinned crystal exhibits a slightly higher flow stress due to bulk dislocation interaction with the twin boundary, at which leading and trailing partials must first recombine into a perfect dislocation in order to cross the twin boundary and glide further.

In the case of $T_{xy}$ loading (Figure 3, top right), the xz plane (Figure 1, left) is sheared in the *x* direction $i_{x}$. In the untwinned crystal (solid black line), this results in stress-strain behavior qualitatively similar to the normal loading cases. In comparison to these latter cases, bulk dislocation nucleation takes place at a lower stress of about 3.8 GPa on $\left(\bar{1}\bar{1}1\right)$. The elastic and dislocation nucleation behavior in the twinned case (dashed red curve) is similar to the untwinned case. In contrast to this latter case, however, bulk dislocation glide in the twinned crystal is again hindered by the (111) twin boundary, resulting in a higher glide stress (dashed red curve) compared to the untwinned case. Note that the twin boundary itself is not affected by $T_{xy}$ loading.

**Figure 4:**
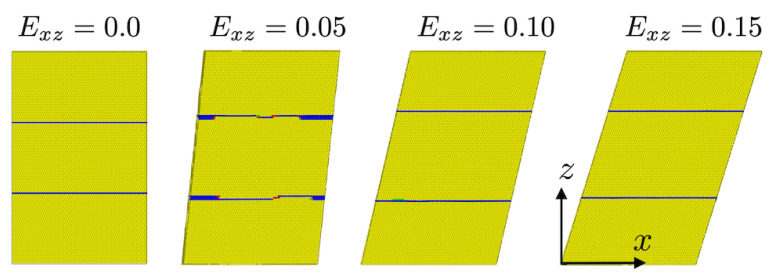
Twin boundary motion and oscillation during $T_{xz}$ loading corresponding to the stress-strain behavior displayed in Figure 3 (middle right).

This is in contrast to $T_{xz}$ (Figure 3, middle right) and $T_{yz}$ (Figure 3, lower right) loading in the xy twin plane. In the former case and untwinned crystal (solid black line), the stress first increases up to about 3.3 GPa and then drops due to homogeneous nucleation of multiple dislocations. The stress increase after $E_{xz}=0.09$ results from the formation of multiple (111) twin regions in the untwinned crystal. At this point, the deformation behavior is governed by twin boundary motion and “jerky” stress flow. The corresponding flow stress is about 2.0 GPa. Comparing this with the stress-strain behavior of the twinned crystal (dashed red line), one sees that the presence of the twin from the start results in no homogeneous dislocation nucleation and corresponding stress drop. Instead, the deformation behavior is determined from the beginning by twin boundary motion, which starts just below 3 GPa and resulting as well in jerky stress flow at a flow stress of about 1.3 GPa. As shown in Figure 4, the motion of the twin boundary is oscillatory, resulting in no further twinning or in detwinning.

**Figure 5:**
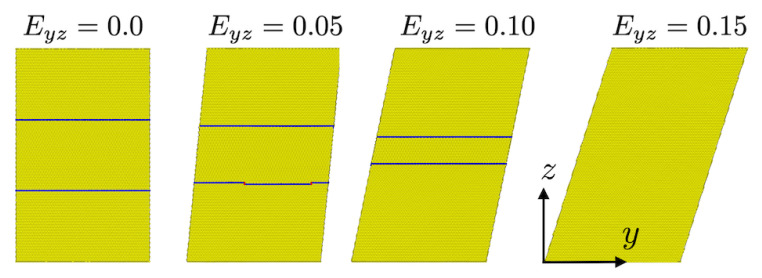
Twin boundary motion and detwinning during $T_{yz}$ loading corresponding to the stress-strain behavior shown in Figure 3 (bottom right).

This is in contrast to the case of $T_{yz}$ loading (Figure 3, bottom right). In the untwinned crystal (solid black line), one observes a stress drop due to homogeneous bulk dislocation nucleation at a much higher yield stress of about 6.2 GPa. The nucleated partials form extrinsic stacking faults and twinned regions. At $E_{yz}=0.10$, the deformation behavior is dominated by partial dislocation motion on the twin boundaries. In the case of the twinned crystal (dashed red line), twin boundary motion begins at just below 3 GPa, similar to the case of $T_{xz}$ loading. Also similar to this latter case is the fact that no homogeneous dislocation nucleation is observed, and the deformation behavior is governed by twin boundary motion. On the other hand, rather than being oscillatory as in $T_{xz}$ case, twin boundary motion under $T_{yz}$ loading leads to detwinning as shown in Figure 5.

At about $E_{yz}=0.14$, the twinned crystal completely untwins, resulting in the linear elastic stress-strain behavior (dashed red curve) shown in Figure 3 (lower right).

**Figure 6:**
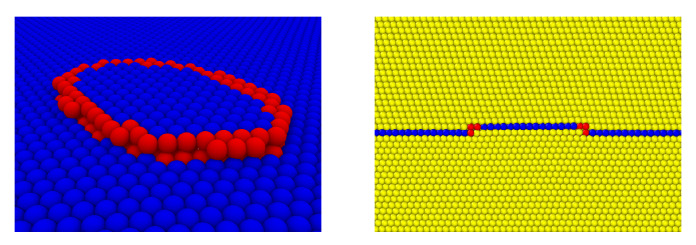
Left: partial dislocation loop (red) on twin plane (blue). Right: cross section of loop and twin plane (blue) in fcc lattice (yellow). As shown, interface partial glide results in normal boundary motion. See text for discussion.

Closer inspection of the serrated or jerky stress-strain behavior, in Figure 3 (middle right, lower right), shows that this is due to fast (111) twin boundary motion resulting from the nucleation and glide of interface or boundary partial dislocations as shown in Figure 6.

**Figure 7:**
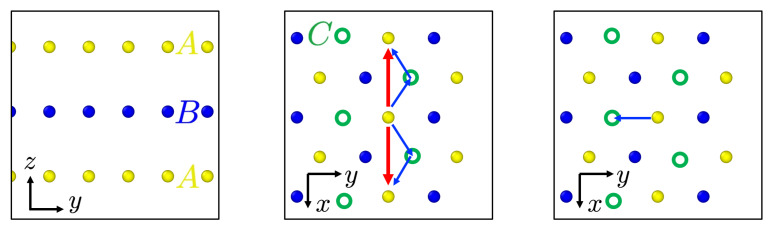
Left: side view of twin boundary (blue) and adjacent (111) planes. Middle and right: top view of the same three atomic layers showing accommodation of $E_{xz}$ (middle) and $E_{yz}$ (right) deformation via dislocation nucleation. Arrows denote perfect (red) and partial (blue) Burgers vectors.

As evident, the twin boundary moves via the nucleation and glide of interface partial dislocation loops along the twin boundary. The different behaviour of twin boundary motion in $T_{xz}$ and $T_{yz}$ loading cases can be explained by the geometry of the twin boundary and adjacent atomic planes as shown in Figure 7.

Consider a positive $E_{xz}$ shear deformation applied to the system. This deformation can be accomodated by perfect dislocation nucleation in the *A* layer (i) above the twin boundary in the positive *x*, or (ii) below the twin boundary in the negative *x*, direction (red arrows, Figure 7, middle). Dislocation nucleation above or below the twin boundary in this case is crystallographically equivalent since the coincident site lattice (CSL) is preserved by both. Consequently, the boundary fluctuates between these two during $T_{xz}$ loading as shown in Figure 4.

In contrast, for $T_{yz}$ loading (Figure 7 right), these are not CSL-equivalent. Indeed, as shown in the figure, a positive *y* displacement of the atoms in the plane above the twin boundary would result in a BB stacking sequence having an unfavorable higher energy. On the other hand, a negative displacement of the atoms in the plane below the twin boundary is more favorable since this results in BC stacking sequence. As such, a positive $E_{yz}$ shear strain will result in partial dislocation nucleation and glide below the twin boundary, and so non-oscillatory translational twin boundary motion leading to further twinning or to detwinning as shown in Figure 5.

The jerky nature of the stress-strain behavior (Figure 3, right middle and bottom) is due to the “fast” motion of the twin boundary in relation to the rate of external loading. This boundary motion results in turn from the nucleation and glide of multiple partial dislocation loops on the twin boundary. Since stress relaxation due to nucleation and glide is faster than the rate of stress increase due to external loading, the stress decreases as the dislocations glide away and the twin boundary moves. Continued external loading then results again in stress increase up to the point at which nucleation occurs again, and the cycle repeats.

### 3.2. Twin Boundary Partial Dislocation Velocity and Energy Barrier to Glide

**Figure 8:**
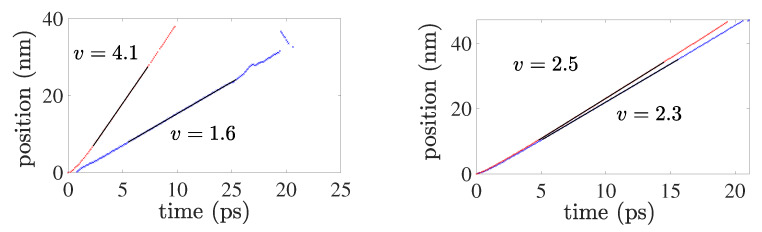
Position as a function of time for edge (**left**) and screw (**right**) partial dislocations in the bulk (blue crosses) and on the twin boundary (red crosses) subject to a load of $T_{xy}=1$ GPa in the edge, and of $T_{zy}=1$ GPa in the screw, case. Position here denotes *x* from Figure 2 in the direction of dislocation line motion $m=i_{x}$. Note that the applied stress induces a Peach-Köhler glide force $f_{\mathrm{gl}}$ of approximately 0.15 N/m along on all dislocations.

As just discussed, nucleation and glide of partial dislocations on the twin boundary result in “fast” twin boundary motion. To investigate this further, the simulation set-up described in Section 2.3 is employed to determine the velocity of partial dislocations on the twin boundary. For comparison, this is also done for the same partial dislocations in the bulk. Figure 8 displays corresponding results based on the set-ups in Figure 2.

**Figure 9:**
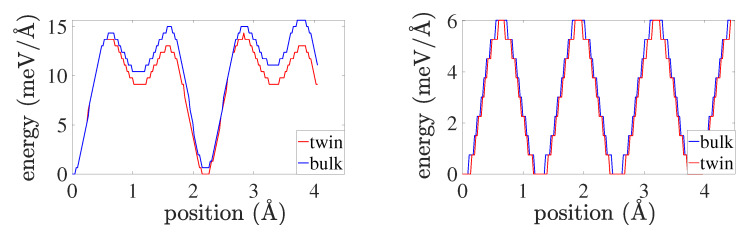
Unrelaxed simulation cell energy per unit dislocation length as a function of dislocation line (core) position for partial edge (**left**) and screw (**right**) dislocations in the bulk (blue) and on the twin boundary (red). As in Figure 8, position denotes *x* from Figure 2 in the direction of dislocation line motion $m=i_{x}$.

As evident, the interface partial edges are faster than the corresponding screws. This is in contrast to the bulk case, for which the partial screws are faster. Results for the associated energy barriers to dislocation glide are displayed in Figure 9.

The energy barrier is calculated using 200 intermediate configurations during the dislocation glide. As shown, the peak interface partial edge energy barrier (i.e., drag) is higher than the corresponding screw barrier by more than a factor of two. Since both are subject to the same loading (i.e., Peach-Köhler glide force $f_{\mathrm{gl}}$), this would seem to be at odds with the higher edge velocity in Figure 8. On the other hand, the energy barrier results for the bulk case are consistent with those for the bulk velocity in Figure 8 in this sense. In the same sense, the velocity (Figure 8) and energy barrier (Figure 9) results for interface and bulk screws are consistent. Note also that the energy barrier to glide for partial screws is lower than that for partial edges; this is in contrast to perfect bulk dislocations, for which the screw barrier is higher than that for edges e.g., [26].

To discuss this in more detail, consider once again the discussion of the Peach-Köhler force above and Figure 2. On fcc octahedral glide planes with $n\equiv \langle 111\rangle /\sqrt{3}$, dislocations have a glide direction m=n×l (recall that $n=i_{y}$ and $m=i_{x}$ in Figure 2). In the case of perfect edge dislocations, for example, we then have $\left(l,m\right)\equiv (\langle 112\rangle /\sqrt{6},\langle 110\rangle /\sqrt{2})$, while for perfect screws, $\left(l,m\right)\equiv (\langle 110\rangle /\sqrt{2},\langle 112\rangle /\sqrt{6})$. For the analogous partial dislocations, note that these are reversed, i.e., $\left(l,m\right)\equiv (\langle 110\rangle /\sqrt{2},\langle 112\rangle /\sqrt{6})$ for partial edges, and $\left(l,m\right)\equiv (\langle 112\rangle /\sqrt{6},\langle 110\rangle /\sqrt{2})$ for partial screws. Since $\langle 110\rangle /\sqrt{2}$ represents a lattice translation vector, but $\langle 112\rangle /\sqrt{6}$ does not, the energy barrier to glide of perfect edges and partial screws is then expected to be lower than that for perfect screws and partial edges. This expectation agrees with the energy barrier results for both the bulk and twin boundary partials in Figure 9.

In summary, the velocity results for bulk partial edge and screw dislocations, as well as for interface and bulk partial screw dislocations, can be explained solely in terms of energy barrier (i.e., drag) effects. On the other hand, this is not the case for the velocity results for interface partial edge dislocations.

### 3.3. Dependence of Deformation Mechanism on Twin Boundary Orientation

**Figure 10:**
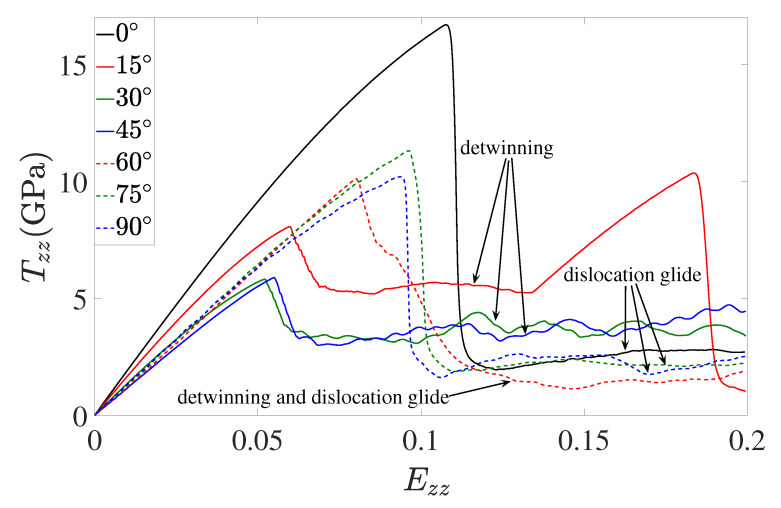
Stress-strain response of the simulation cell in Figure 1 (right) for θ values of $0^{{}^{\circ}}$, $15^{{}^{\circ}}$, $30^{{}^{\circ}}$, $45^{{}^{\circ}}$, $60^{{}^{\circ}}$, $75^{{}^{\circ}}$, $90^{{}^{\circ}}$ subject to $T_{zz}$ loading. Again, all other stress components are zero.

The following results are based on the simulation cell in Figure 1 (right) with twin boundary orientation θ subject to loading in *z* direction (i.e., $T_{zz}$ loading). Corresponding stress-strain results are shown in Figure 10.

In particular, for $\theta =0^{{}^{\circ}}$ (solid black line), loading is normal to the twin boundary. Consequently, the observed stress drop and relaxation above 10% strain and 15 GPa is due to bulk dislocation nucleation and glide. For $\theta =15^{{}^{\circ}}$, $30^{{}^{\circ}}$, $45^{{}^{\circ}}$ (solid red, green and blue lines, respectively), $T_{zz}$ loading results in resolved shear stress on the twin boundaries, driving twin boundary motion and detwinning. The corresponding yield stresses and stress drops are much smaller than for $\theta =0^{{}^{\circ}}$ since dislocation nucleation is limited to the twin boundary and interface partials, in contrast to the homogeneous nucleation of bulk dislocations for $\theta =0^{{}^{\circ}}$. Note also the reappearance of perfect lattice stress-strain behavior (solid red line) after detwinning for $\theta =15^{{}^{\circ}}$ in Figure 10, analogous to the case in Figure 3 (bottom right). For $\theta =75^{{}^{\circ}}$, $90^{{}^{\circ}}$, twin boundary motion is reduced, and bulk dislocation glide becomes the dominant deformation mechanism. Excepted for the reduced yield stress, then, these case tend toward the pure normal loading case $\theta =0^{{}^{\circ}}$. In both cases, the twin boundary is not moving. Note that $90^{{}^{\circ}}$ and $0^{{}^{\circ}}$ are equivalent to $T_{yy}$ and $T_{zz}$ in Figure 3, respectively.

**Figure 11:**
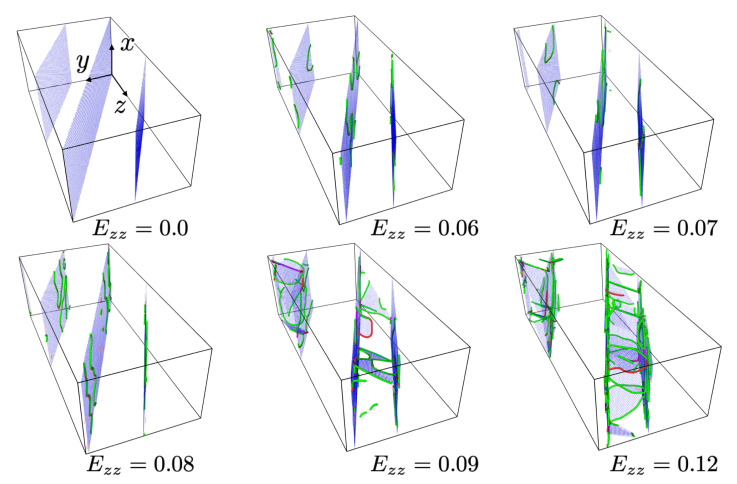
Combined twin boundary motion and bulk dislocation nucleation-slip for $\theta =60^{{}^{\circ}}$ with increasing loading. Blue: twin boundaries and stacking faults. Red: dislocation lines. The dislocations are extracted using the dislocation extraction analysis (DXA) tool in Ovito. For better visualization, fcc lattice atoms have been removed.

For the case $\theta =60^{{}^{\circ}}$, both twin boundary motion and bulk dislocation nucleation-slip are observed, as shown in Figure 11.

Initially, partial dislocation motion on the twin boundary results in twin boundary motion (from $E_{zz}=0.06$ to $E_{zz}=0.08$). However, as the load increases, deformation of the simulation cell results in a change in the angle between the loading direction and the twin boundary orientation up to around $E_{zz}=0.09$, at which point bulk dislocations nucleate and begin to glide. More specifically, as the system elongates in the *z* axis during loading, the twin boundaries rotate toward the *z* axis (see Figure 11), resulting in an effective increase of θ and a shift from interface to bulk dislocation nucleation and glide as the effective shear stress on the twin boundaries decreases. As shown in Figure 11, bulk dislocations nucleated in form of partial dislocations with extended stacking faults. They glide and interact with the twin boundaries, resulting in bulk slip-twin interaction and bulk slip transfer across the twin boundaries. Based on the simulation results, the very first bulk dislocation is nucleated at $E_{zz}=0.083$ and an effective θ of about 68°.

## 4. Summary and Conclusions

In this work, the effect of twinning on the bulk mechanical behavior of fcc Cu is studied using molecular dynamics. Several tensile, shear, and mixed, loading cases are investigated and the results are compared with the reference stress-strain behavior of an untwinned fcc crystal. In particular, shear loading of the twin boundary results in significantly different behavior than in the other loading cases, and in particular to jerky stress flow. More specifically, twin boundary shear loading along 〈112〉 results in translational normal twin boundary motion, twinning or detwinning, and net hardening. On the other hand, such loading along 〈110〉 results in oscillatory normal twin boundary motion and no hardening. As documented in the current work, jerky flow and stress fluctuation due to twin boundary motion are related to fast partial dislocation nucleation and glide on the twin boundary.

Velocity results for bulk partial edge and screw dislocations, as well as for interface and bulk partial screw dislocations, can be explained solely in terms of energy barrier (i.e., drag) effects. On the other hand, this is not the case for the velocity results for interface partial edge dislocations. Indeed, given that both are subject to the same loading, one would expect the higher energy barrier to glide for twin boundary partial edges (Figure 9) to result in a lower velocity (Figure 8) than for twin boundary partial screws (i.e., in Cu at 20 K). Instead, the velocity results for twin boundary straight partial edge and screw dislocations (Figure 8) show that partial edges move almost twice as fast as partial screws.

Apparently, additional mechanisms or atomistic effects are involved here beyond short-range energy barrier and/or long-range (continuum) Peach-Köhler considerations. More accurate determination of the energy barriers based for example on nudged elastic band and/or DFT information may shed further light on this. In any case, this represent on-going work and research in progress to be reported on in the future.

For the case of variable twin boundary orientation in relation to the loading direction, a strong dependence of dominant deformation mechanism is observed as the angle θ between the twin plane normal and loading direction increases from 0 to 90 degrees. In particular, this includes a transition in dominant deformation mechanism from (i) bulk dislocation slip/stationary twin boundary interaction ($\theta =0^{{}^{\circ}}$) to (ii) twin boundary motion (twinning, detwinning) ($\theta =15^{{}^{\circ}},30^{{}^{\circ}},45^{{}^{\circ}}$) to (iii) mixed twin boundary motion, bulk slip/slip transfer ($\theta =60^{{}^{\circ}}$) to (iii) bulk slip/twin boundary interaction ($\theta =75^{{}^{\circ}},90^{{}^{\circ}}$). This is in agreement with previous work.

Besides clarification of the velocity behavior of twin boundary partial edge dislocations discussed above, future work includes extension of the current study to variable temperature, other fcc materials, and to finite systems including for example interaction with grain boundaries and other defects.
